# Long-Term Monitoring of Rainfed Wheat Yield and Soil Water at the Loess Plateau Reveals Low Water Use Efficiency

**DOI:** 10.1371/journal.pone.0078828

**Published:** 2013-11-26

**Authors:** Wei Qin, Baoliang Chi, Oene Oenema

**Affiliations:** 1 Dryland Agriculture Research Centre, Shanxi Academy of Agricultural Sciences, Taiyuan, Shanxi, China; 2 Environmental Sciences, Wageningen University, Wageningen, The Netherlands; University of Saskatchewan, Canada

## Abstract

Increasing crop yield and water use efficiency (WUE) in dryland farming requires a quantitative understanding of relationships between crop yield and the water balance over many years. Here, we report on a long-term dryland monitoring site at the Loess Plateau, Shanxi, China, where winter wheat was grown for 30 consecutive years and soil water content (0–200 cm) was measured every 10 days. The monitoring data were used to calibrate the AquaCrop model and then to analyse the components of the water balance. There was a strong positive relationship between total available water and mean cereal yield. However, only one-third of the available water was actually used by the winter wheat for crop transpiration. The remaining two-thirds were lost by soil evaporation, of which 40 and 60% was lost during the growing and fallow seasons, respectively. Wheat yields ranged from 0.6 to 3.9 ton/ha and WUE from 0.3 to 0.9 kg/m^3^. Results of model experiments suggest that minimizing soil evaporation via straw mulch or plastic film covers could potentially double wheat yields and WUE. We conclude that the relatively low wheat yields and low WUE were mainly related to (i) limited rainfall, (ii) low soil water storage during fallow season due to large soil evaporation, and (iii) poor synchronisation of the wheat growing season to the rain season. The model experiments suggest significant potential for increased yields and WUE.

## Introduction

Water scarcity is a growing global concern [1]–[4]. For rainfed agriculture, this pressure may become more severe under climate change due to the expected more erratic rainfall and longer dry spells [5]–[8]. Currently, rainfed agriculture covers 80% of the world’s cultivated land and accounts for 60% of crop production [9]. However, crop yield and water use efficiency (WUE) are often low in rainfed agriculture, especially in arid and semi-arid areas due to, for example, degraded soils, erratic rainfall and poor water management [10]. Many of these areas in Africa and Asia face also rapid population growth. Hence, there is a pressing need to increase crop yields and WUE in rainfed agriculture [11]–[15].

Water use efficiency is commonly defined as crop yield over evapotranspiration (ET), where ET is the sum of soil evaporation (E) and crop transpiration (T) [16]–[19]. The latter (T) is a direct consequence of crop production, while E is ‘unproductive’ water loss. The central question for rainfed agriculture in arid and semi-arid regions is ‘how to transform unproductive water loss (E) into productive water use (T)’. Unfortunately, the partitioning between E and T is often not well-known, due to difficulties and high cost in distinguishing E and T in the field. As a result, water use of crops is commonly reported as evapotranspiration (ET) [16], [17], [20]. This lack of information makes it difficult to assess how much of the evaporative water loss can be used for increasing yields by appropriate measures.

Crop growth simulation models have the potential of providing more comprehensive insights into the functioning of soil-crop systems, and can be helpful to explore options for increasing yield and WUE [21]–[23]. These models though are simplified representations of parts of reality and therefore require testing in the real world. Fortunately, numerous field studies have examined the effect of water availability, with or without irrigation treatments, on crop yield and WUE [16]–[20]. In principle, these results can be used to calibrate and test the crop growth simulation models. However, most of these field studies are short-term (2–5 years) and focus on the growing season only, largely ignoring the water balance during the fallow season. Furthermore, crop yields and WUE show large variations due to differences in soils, climate conditions and crop husbandry practices. For example, in the North China Plain, annual precipitation ranges from 400 to 650 mm and wheat grain yields roughly range from 1 to 3 ton/ha/year under rainfed conditions. With 200 to 300 mm of irrigation, grain yield can be increased by 60 to 100% and WUE can be increased by 20 to 40% [16]. Globally, WUE of wheat shows even larger variation ranging from approximately 0.2 to 1.8 kg/m^3^ [24]. Evidently, this wide range suggests considerable scope for improvement, but the underlying causes of WUE is not always well-known and irrigation water is not available on most places.

The study reported here has the objectives (i) to calibrate a water-driven crop growth model on the basis of monitoring data from a long-term field experiment with rainfed winter wheat, (ii) to analyse the components of the water balance of this field, and (iii) to explore the potential for increasing crop yield and WUE through model experiments. The field experiment was situated in the dryland of the Loess Plateau, Shanxi, China. Winter wheat was grown for 30 consecutive years and soil water content (0–200 cm) was measured every 10 days. The FAO AquaCrop model was chosen as simulation model because it is a water-driven crop growth model that can separately calculate E and T, and simulates the final crop yield as function of water use [23], [25]. The AquaCrop model uses canopy ground cover as the basis to calculate T and to separate E and T. Crop yield is then calculated as the product of biomass and harvest index (HI). The principles and modules of the AquaCrop model are well-documented in a series of AquaCrop publications [23], [25]–[28]. Compared to some other crop growth models, AquaCrop requires relatively few input parameters [23], [28]. Such a limited number of input parameters facilitates model calibration and utilization for different crops and under different management strategies [23], [26], [27], [29]–[36].

## Materials and Methods

### Site Information

The long-term monitoring site is located in Beizhang, Linyi county in Shanxi province on the Loess Plateau (35° 9′ 3.83″ N, 110° 34′ 25.40″ E, Altitude: 491 m) in China. The authority is Dryland Agriculture Research Centre, Shanxi Academy of Agricultural Sciences. We have the permission to conduct the study on this site. We confirm that the field studies did not involve endangered or protected species.

The site has a semi-arid climate with extensive monsoonal influence, which is dry and cold in winter, rainy and hot in summer. Rainfall in June to September accounts for more than 70% of annual rainfall. Average annual rainfall was 517 mm in the period of 1980–2010 with large annual variations, from a minimum of 331 mm to a maximum of 832 mm. Mean annual sunshine duration is 2270 h, annual average temperature 13.5°C and mean annual potential evaporation is 1340 mm. The soil is a typical Loessial soils (Calcic Luvisols) of the Loess Plateau [37]–[40]. Soil slope is <1% and soil texture is silt loam, with a small proportion of clay. The soil was rather homogeneous in texture and key physical properties (Table 1). Maximum soil water holding capacity was a significant fraction of the total annual rainfall (Table 2).

**Table 1:** Soil physical properties for different layers up to 200

Soil layer	Bulk density	Field Capacity	Wilting point	Texture, (particle size, µm in %)			
cm	g/cm^3^	v/v in %	v/v in %	>63 µm	63–20 µm	20–2 µm	<2 µm
5	1.34	31.4	6.8	7.5	41.0	32.4	19.2
10	1.34	31.4	6.8	7.5	41.0	32.4	19.2
20	1.39	32.9	8.5	8.3	41.8	31.2	18.8
30	1.43	34.5	8.4	6.9	44.3	30.1	18.7
40	1.39	33.6	7.0	6.9	44.3	30.1	18.7
50	1.43	33.6	7.3	6.9	44.3	30.1	18.7
60	1.39	32.8	7.0	4.5	40.5	31.4	23.6
70	1.36	30.9	7.2	4.5	40.5	31.4	23.6
80	1.25	27.6	6.9	4.5	40.5	31.4	23.6
90	1.28	27.9	8.3	4.5	40.5	31.4	23.6
100	1.32	28.3	8.2	4.5	40.5	31.4	23.6
120	1.30	29.3	7.8	3.0	43.4	34.4	19.2
140	1.32	29.7	7.9	3.0	43.4	34.4	19.2
160	1.31	29.5	7.9	2.4	40.4	38.7	18.5
180	1.32	29.7	7.9	2.4	40.4	38.7	18.5
200	1.32	29.7	7.9	2.4	40.4	38.7	18.5

**Table 2:** Soil moisture holding capacity of the soil profile (0–200 cm).

	Total water, mm(mm)	Available water, mm(mm)	In volume, % (v/v)
**Saturation**	860	711	43
**Field capacity**	596	447	30
**70% FC**	462	312	23
**50% FC**	373	223	19
**Wilting point**	149	0	8

The top soil (30 cm) contained 5.8 g/kg of organic C and 0.55 g/kg of total N. Mean available N, P and K were 63 mg/kg, 14 mg/kg and 142 mg/kg, respectively, in 2007. Since 1983, mean available P and K have been increased by 7 mg/kg and 10 mg/kg, respectively [41]. Total soil organic C was measured by dry combustion combined with elemental C analysis. Total N was measured by Kjeldahl method. Soil mineral N was extracted with 1 M KCl and analysed by the cadmium reduction method. Available P was extracted with 0.5 M NaHCO_3_. Available K was extracted with 1.0 M NH4OAc.

### Experimental Design and Measurements

**Figure 1:**
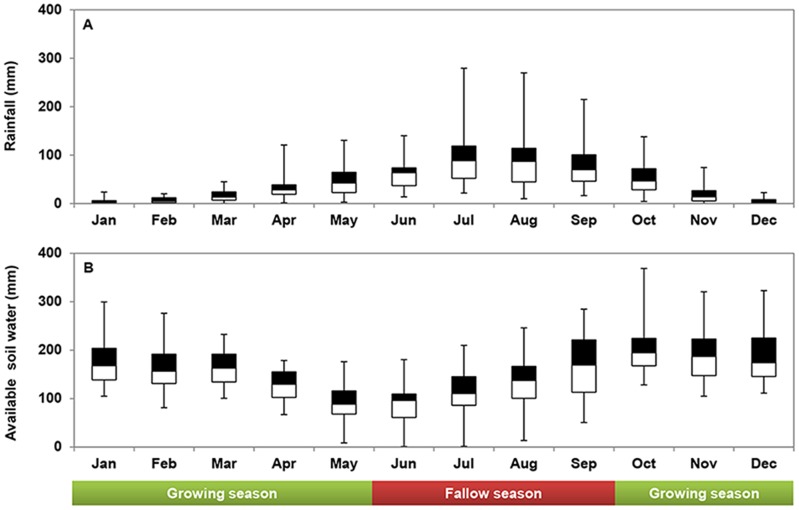
Monthly rainfall and available soil water during the period 1980–2010. A: monthly rainfall distribution; B: monthly available water content. Black and white boxes show 75 and 25% percentile values. Whiskers show maximum and minimum values (the same applies to other figures). The growing season of winter wheat was 245 days from 1 October to 1 June. of the next year, highlighted in green bar. Fallow season was 120 days from 2^nd^ Jun. to 30^th^ Sep., highlighted in red bar.

**Figure 2:**
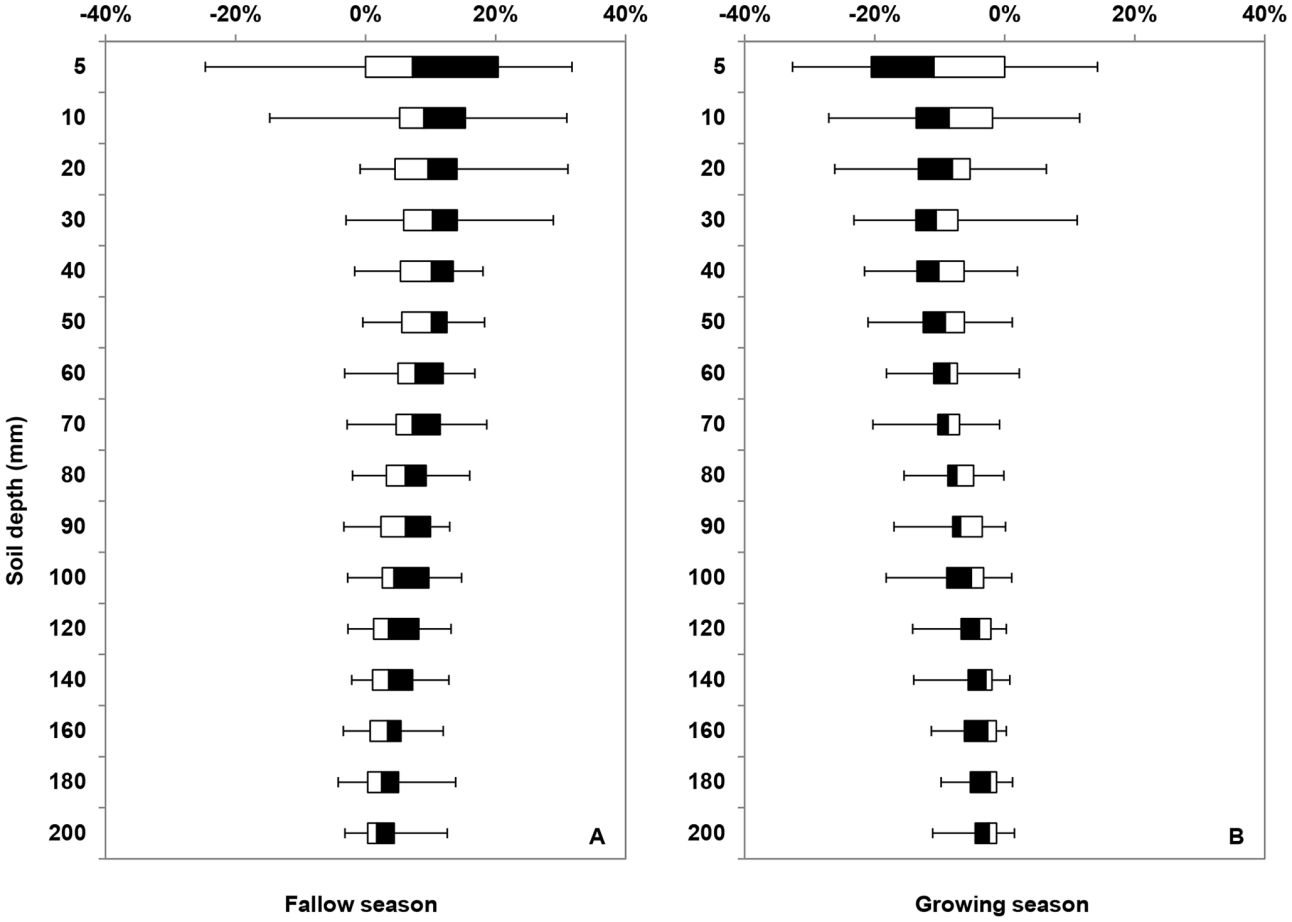
Changes in soil water content in the upper 2 meter of the soil during the period of 1980–2010. A: water stored in the upper 2 meter during the fallow season, presented in positive percentage (in volume, v/v). B: water depletion in the upper 2 m during the growing season, presented in negative percentage (in volume, v/v). The soil water changes for fallow season were calculated as the soil water content of October deducted by that of June. Similarly, the soil water changes for growing season were calculated as the soil water content of June deducted by that of October in the previous year.

The long-term crop yield and soil moisture monitoring experiment started in 1980. There were no experimental treatments. The size of the field is 0.5 ha. Winter wheat was planted each year between 25 September and 5 October, depending on the actual climate and soil water conditions. Sowing rate was 150 kg seed/ha. The growing period of the winter wheat is approximately 245 days (from 1 October to 1 June of the next year). The fallow season is about 120 days (from 2^nd^ June to 30^th^ September) (Figure 1). Local bred cultivars (Jinmai) were used for all years. Information about crop parameters is listed in Table 3. Fertilizers were applied at planting at a rate of 127.5 kg/ha of N as urea and 90 kg/ha of P_2_O_5_ as superphosphate, for all years. At harvest, grain yields were measured in 5 random plots (2 m^2^ per plot) selected from two diagonal lines of the field. Dry matter content of grain and straw was measured at the laboratory after drying at 70°C. Soil water contents were measured in 16 different layers up to 2 m depth every 10 days by using the gravity method. The top 10 cm was sampled in 5 cm intervals. From 0.1 to 1 m, samples were taken at 10 cm intervals, and from 1 to 2 m at 20 cm intervals (Figure 2). Each sample consisted of 5 subsamples, taken randomly on two diagonal lines across the field. Observations during soil sampling over years revealed that spatial variations in soil profile were small.

**Table 3:** Full set of crop parameters used in this study.

Parameters	Contents and values	Source
**Crop development**	in calendar days	
From sowing to emergence	7 days	Field observation
From sowing to max. canopy	200 days	Field observation
From sowing to flowering	200 days	Field observation
From sowing to senescence	210 days	Field observation
From sowing to maturity	245 days	Field observation
Length for building up Harvest Index (HI)	44 days	Field observation
Duration of flowering	5 days	Field observation
Plant density	300 plants/m^2^	Field observation
Sowing rate	150 kg seed/ha	Field observation
1000 seed mass	40 g	Field observation
Germination rate	80%	Field observation
Max. root depth	2 m	Field observation
Reference HI	42%	Field observation
Max HI	48%	Field observation
Crop water productivity	15 g/m^2^	Calibrated
**Canopy development**		
Initial canopy cover	4.5%	Field observation
Canopy expansion	2.9%/day	Calculated by AquaCrop
Max. canopy cover	90%	Field observation
Canopy decline	7.2%/day (39 days)	Calculated by AquaCrop
**Thresholds temperatures**		
Base temperature for biomass production	0°C	Calibrated
Upper temperature for biomass production	26°C	Calibrated
Range of cold stress for biomass production	0–14°C	Calibrated
Range of cold stress for pollination	4–9°C	Calibrated
Range of heat stress for pollination	32–37°C	Calibrated
**Water extraction pattern throughout** **the root zone**		
Upper 1/4 (0–0.5 m)	40%	Calibrated
Second 1/4 (0.5–1 m)	30%	Calibrated
Third 1/4 (1–1.5 m)	20%	Calibrated
bottom 1/4 (1.5–2 m)	10%	Calibrated
**Water stresses**		
Canopy expansion	Moderately tolerant (upper = 0.25, lower = 0.65, shape factor = 5)	Calibrated
Stomatal closure	Extremely sensitive (upper = 0.25, shape factor = 2.5)	Calibrated
Early canopy senescence	Tolerant (upper = 0.75, shape factor = 2.5)	Calibrated
Aeration stress	Moderately tolerant (5 vol%)	Calibrated
**Evapotranspiration**		
Soil evaporation coefficient	Effect of canopy shelter in late season = 50%	AquaCrop default value
Crop transpiration coefficient	1.1 (reduction with age = 0.15%/day)	AquaCrop default value
**Fertilities stresses**	Not considered	AquaCrop default value

### Water Balance and WUE Estimations

Mean monthly rainfall data were collected at a near-by meteorological station (Linyi station, 35° 10′ 7.02″ N, 110° 46′ 44.59″ E, Altitude: 441 m), which is 25 km away from the experimental field. The water balance for both fallow and crop growing seasons reads as follows:
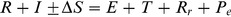
(1)where R is rainfall, I is irrigation, ΔS is the change of soil water content, E is soil evaporation, T is crop transpiration, R_r_ is runoff and P_e_ is percolation. All units are presented in mm or in m^3^/ha.

Irrigation was not applied in this study. Furthermore, runoff and percolation (leaching) were small and disregarded. Hence, the water balance of the fallow seasons was simplified to:
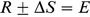
(2)


The water balance of the growing season was simplified to:
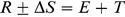
(3)


We used formula (2) and the recorded rainfall and soil water content data to calculate evaporation during the fallow season and formula (3) to calculate ET during the growing season. The partitioning of E and T was done by the AquaCrop model.

Water use efficiency (WUE, kg/m^3^) was calculated as:
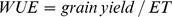
(4)where ET is evapotranspiration (mm), the sum of E and T during the growing season.

### Calibration and Validation of the AquaCrop Model

The FAO AquaCrop model is a water-driven crop growth model for the simulation of crop biomass and yield as function of water availability [23], [25]. AquaCrop requires 4 main sets of input data, i.e. climate data (rainfall, minimum and maximum temperature and reference evapotranspiration (ETo)), crop parameters, soil data and field management data. Climate data were collected from a near-by meteorological station (Linyi station, 35° 10′ 7.02″ N, 110° 46′ 44.59″ E, Altitude: 441 m), which is 25 km away from the experimental field. ETo was calculated by FAO Penman-Monteith equation as described in Allen et al. [42]. Soil data (Tables 1 and 2) and field management data were derived from measurements and from recordings at the monitoring site. Crop data and parameters were derived from measurements, literature [23], [25]–[27] and by calibration (Table 3).

The objective of calibration and validation was to achieve the best match between simulated outputs and monitoring data (soil moisture and crop yield) for all 30 years, using a common procedure [27], [29]–[36]. We randomly selected 15 years’ data from the monitoring field for calibration and used the other 15 years’ data for validation of the calibrated model. In the calibration step, we used the year-specific climate, soil and initial soil water data as fixed input data. Then we adjusted some of the crop parameters (see Table 3), based on our understanding of crop growth, development and crop responses to water deficits, until the differences between simulated output and monitoring data were minimal. We repeated this process for all selected 15 years and ultimately obtained a satisfactorily index of agreement (d = 0.92). Then, the calibrated model was validated with the other 15 years’ data, and again obtained an acceptable index of agreement (d = 0.93).

### Data Analysis

The root mean square error (RMSE) [43] has been widely used to evaluate the performance of a model [30], [44]–[47]. However, Willmott and Matsuura [44] pointed out that, compared to RMSE, the mean absolute error (MAE) is a better indicator and therefore the evaluation of model performance should be based on the MAE. In this study, we used the MAE, the mean bias error (MBE) and the Willmott index of agreement (d) to evaluate the model performance.

The MAE measures the weighted average magnitude of the absolute errors and was calculated as follows:
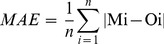
(5)where n is the number of observations, Mi is the modelled yield or soil water content and Oi is the observed yield or soil water content.

The MBE indicates whether the model is under or over predicting the observed values and also indicates the uniformity of error distribution. Positive MBE values indicate over prediction, negative values indicate under prediction and a value of zero indicates equal distribution between negative and positive values. The MBE was calculated as follows:
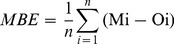
(6)


The Willmott index of agreement (d) [43] has values ranging from 0 to 1. A value close to 1 suggests a good model performance. The agreement index d was calculated as follows:
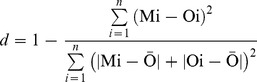
(7)where 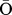 is the average value of the observed yield or soil water content.

### Model Experiments

To investigate the potentials of minimizing soil evaporation so as to increase crop yield and WUE, we set up six model experiments as follows:

Experiment 1 (E1): Reference A; winter wheat was planted at 50% of field capacity (FC) (low soil water content)

Experiment 2 (E2): E1+organic mulch (straw)

Experiment 3 (E3): E1+plastic film cover

Experiment 4 (E4): Reference B; winter wheat was planted at 70% of FC (high soil water content)

Experiment 5 (E5): E4+organic mulch (straw)

Experiment 6 (E6): E4+plastic film cover

In the model experiments, we used E1 and E4 as references to simulate crop growth under relatively low (with 50% FC) and high (with 70% FC) soil water content at winter wheat seeding (Table 2). The range from 50 to 70% FC largely represented the initial soil water content for the planting period. The low value is representative for no water harvesting, and the high values is representative for water harvesting during the fallow period via mulching and/or covers. Model experiments E2 and E5 aimed at testing the effects of organic mulch during the growing season, and experiments E3 and E6 aimed at testing the effects of plastic film during the growing season on crop yield and water balance. The effectiveness of the soil evaporation reduction by organic mulch and plastic film cover during the growing season were estimated at 50 and 90%, respectively, which are default values of the AquaCrop model [25]. However, since plastic film will cover only about 80% of the wheat planted field, the overall soil evaporation reduction by plastic film was set at 72%. In practice, the plastic film covers are used for the growing season only and destroyed or removed after harvesting the crops. We ran these six experiments for the period of 1980–2010.

## Results

### Patterns of Monthly Rainfall and Soil Water Content

Most of the rainfall occurs in the summer from June to September, accounting for more than 70% of the total annual rainfall while winter wheat was seeded in the end of September or beginning of October, and harvested in the end of May or beginning of June. Hence, most of the rain fell when wheat had matured already, i.e., during the fallow period. Therefore, the growing season of winter wheat was poorly synchronized to the rain season. On the other hand, due to concentrated rainfall in June to September, mean soil water content in the upper two metre was highest in October when winter wheat was seeded. Thereafter, the soil water content gradually decreased, due to soil evaporation and crop transpiration. Mean soil water content was lowest in June, at the beginning of the rain season when the winter wheat was harvested (Figure 1).

The changes of the soil water content (for 16 layers in the upper 2 meter of the soil) during the fallow and growing seasons are shown in Figure 2. The changes in water content in the fallow season mirror the changes during the growing season, i.e. water stored in the soil during the fallow season largely equalled to soil water depletion during the growing season. The soil water changes for fallow season were calculated as the soil water content of October deducted by that of June. Similarly, the soil water changes for growing season were calculated as the soil water content of June deducted by that of October in the previous year. Soil water content mostly changed in the top 1 m, accounting for 70% of total soil water change (±120 mm) (Figure 2). We cannot exclude that some of the seasonal variations are caused by slight spatial variations in soil characteristics.

### Model Calibration and Performance

**Figure 3:**
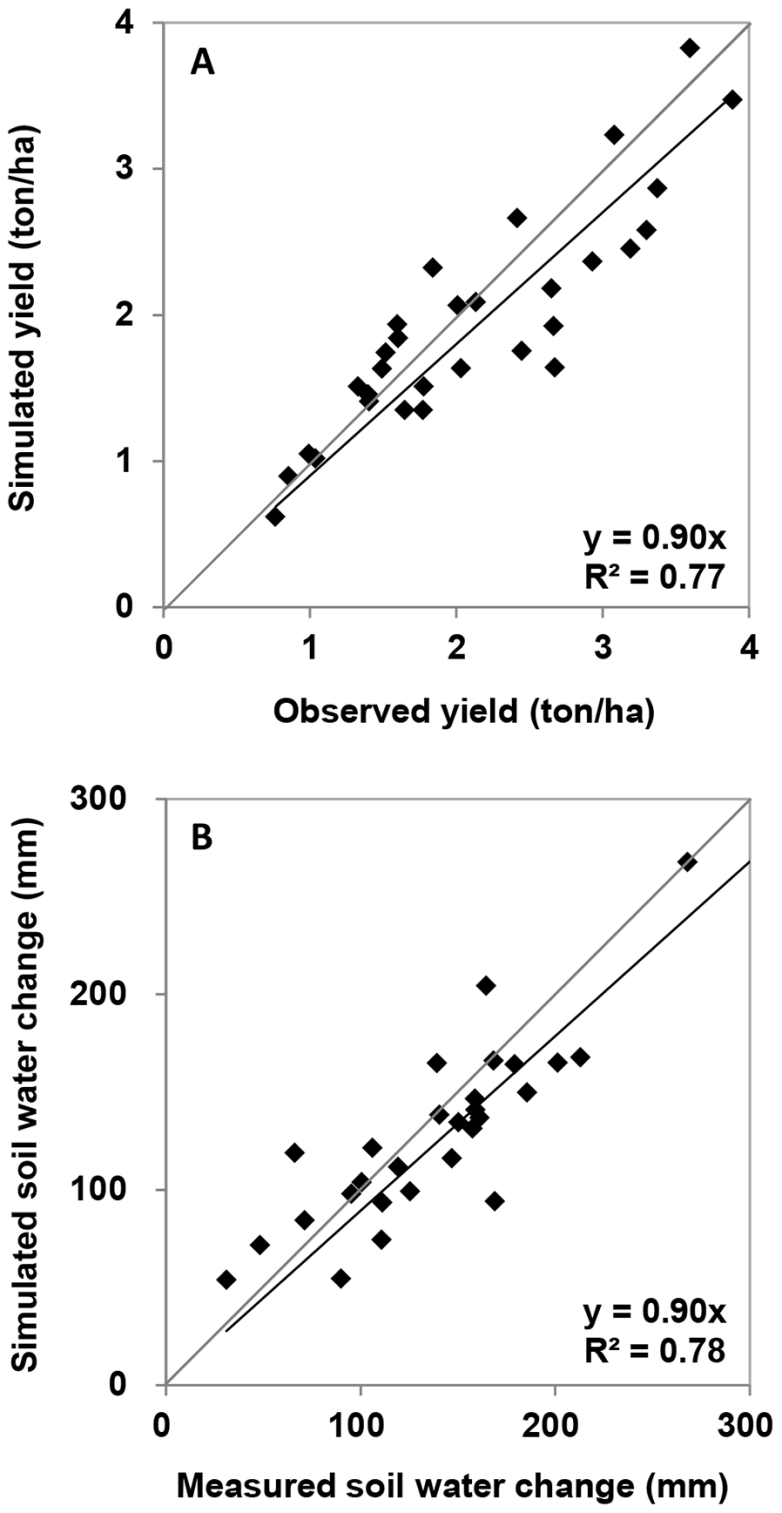
The AquaCrop Model simulations on yield (A) and soil water change (B). Diagonal lines represent 1∶1 lines.

The AquaCrop model was calibrated using the climate data, soil data, field management data and monitoring data. The full set of crop parameters is listed in Table 3. The performance of the model on the simulated crop yield and soil water balance is shown in Figure 3. The relationship between observed and modelled grain yield of the calibrated model for the second set of 15-years data (validation step) was almost as good as for the whole set of data (not shown). For the 30 years’ data, the relationship between observed and modelled grain yield had a correlation coefficient of (R^2^) of 0.77, and the index of agreement (d) was 0.93 (Figure 3A). Similarly, the relationship between observed and modelled soil water content showed a R^2^ of 0.78 and an index of agreement (d) of 0.93 (Figure 3B). Mean absolute errors (MAE) were 311 kg/ha and 25 mm, respectively. Mean bias errors (MBE) were −168 kg/ha and −12 mm in simulating yields and soil water balance, respectively, suggesting that the model slightly underestimated grain yields and soil water contents by 8 and 9%, respectively. We conclude that the performance of the AquaCrop model was acceptable for doing further simulations.

### Water Balance and Partitioning of E and T

**Figure 4:**
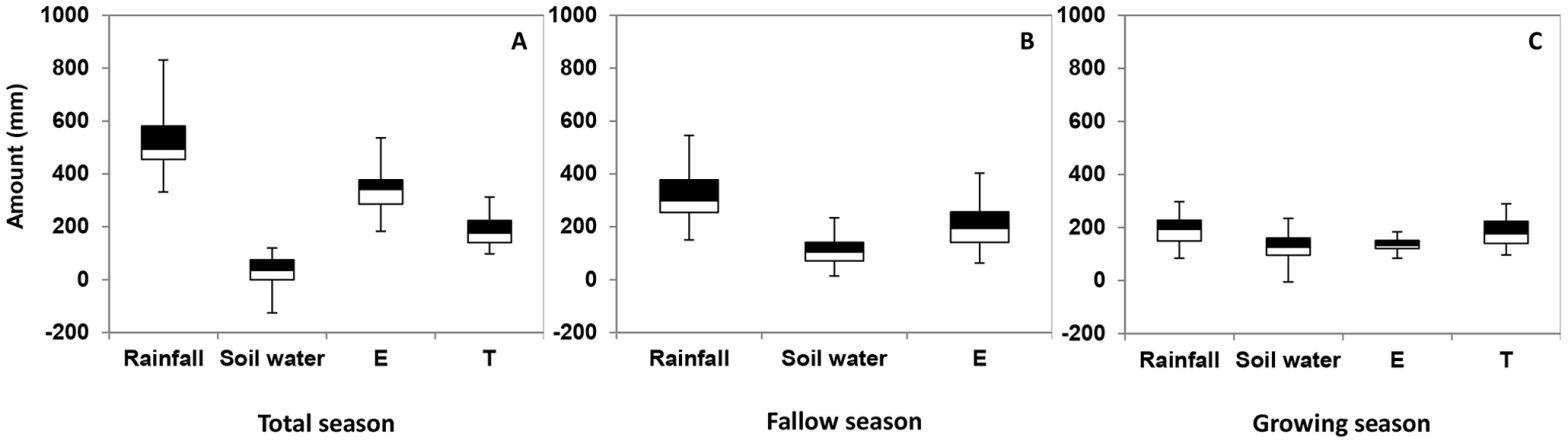
Water balance (rainfall, change in soil water, E, T) of the total season (A), fallow season (B) and growing season (C) during the period of 1980–2010. Total season means the sum of fallow season and growing season.

AquaCrop was used to estimate soil evaporation and crop transpiration, and the water balance for both fallow and growing seasons during the period 1980–2010 (Figure 4). There was only a very marginal change in soil water content when considering the water balance of the total season (fallow+growing season) over the 30 years’ period; total rainfall was nearly equal to the sum of soil evaporation and crop transpiration. Crop transpiration accounted for approximately one-third of total seasonal rainfall. The remaining two-thirds were assumed to be lost by soil evaporation (Figure 4A), but we cannot exclude that a small fraction of this evaporative loss was actually lost by leaching and/or runoff. Mean rainfall in the fallow season was 323 mm, of which 207 mm (64%) was lost by soil evaporation and 116 mm (36%) was stored in the soil (Figure 4B). During the growing season, mean crop transpiration was 185 mm (57%) and soil evaporation was 137 mm (43%), of which rainfall and soil moisture contributed 194 (60% ) and 128 mm (40%), respectively (Figure 4C). Mean rainfall of the growing season was slightly larger than mean crop transpiration.

### Wheat Yield and WUE

**Figure 5:**
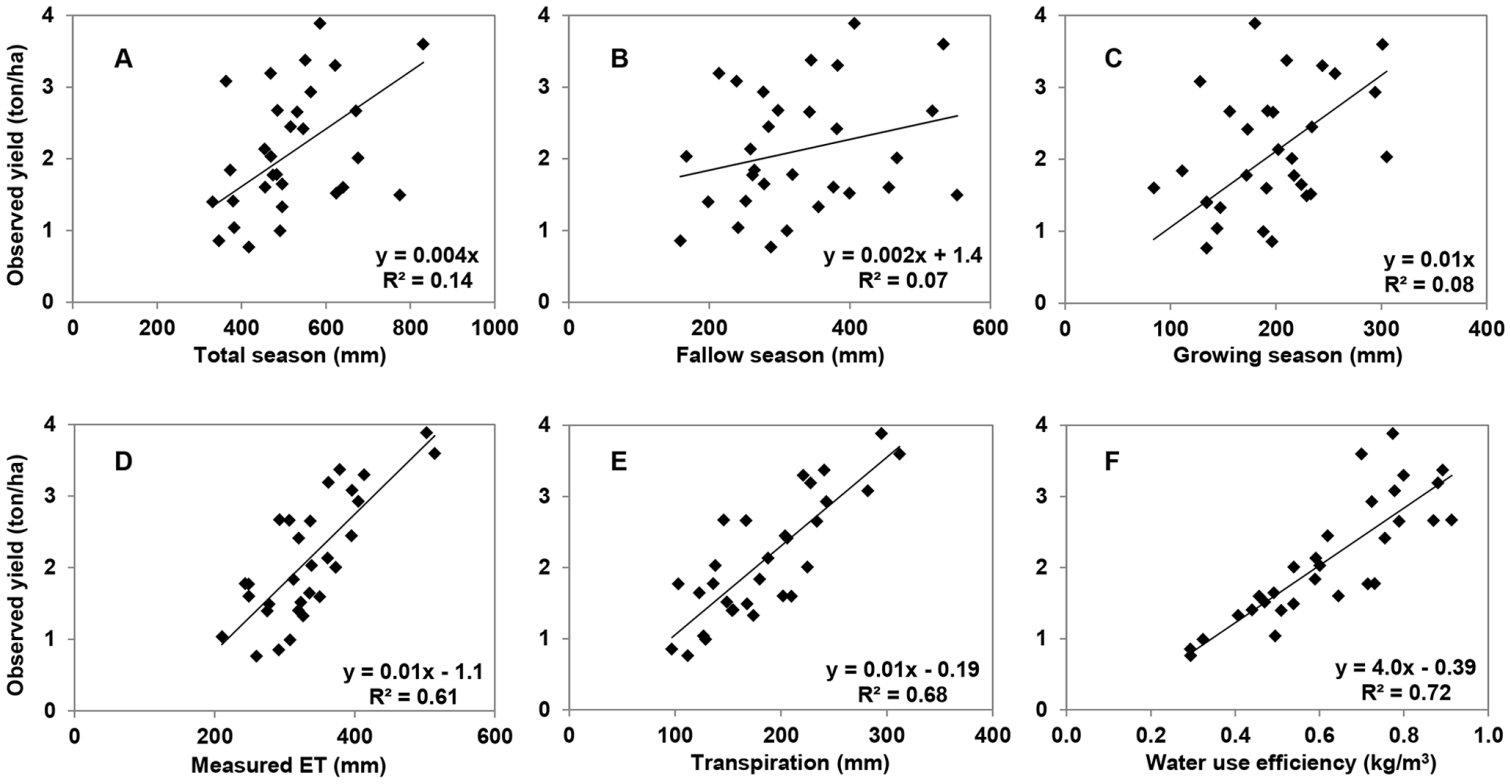
Relationships between observed yield and total rainfall (A), rainfall in fallow season (B), rainfall in growing season (C), measured ET (D), Transpiration (E) and WUE (F). The significant level is 0.028 for (A), 0.167 for (B), 0.027 for (C). For D, E and F, the significant level is all smaller than 0.01.

Due to limited amounts of available water and the irregular rainfall pattern, wheat yield and WUE were rather low, ranging from 0.6 to 3.9 ton/ha/year and from 0.3 to 0.9 kg/m^3^, respectively. Relationships between yield and annual rainfall (p = 0.028), and between yield and growing season rainfall (p = 0.027) were highly significant (Figures 5). The relationship between yield and rainfall during the fallow season was not significant (p = 0.167). Furthermore, there were significant linear relationships between wheat grain yield and ET, T, and WUE, with coefficients of determination (R^2^) of 0.61, 0.68 and 0.72, respectively (Figure 5). Given the slope (0.01) between yield and T, an increase of 1 mm available water can produce 10 kg grain per hectare.

### Model Experiments

**Figure 6:**
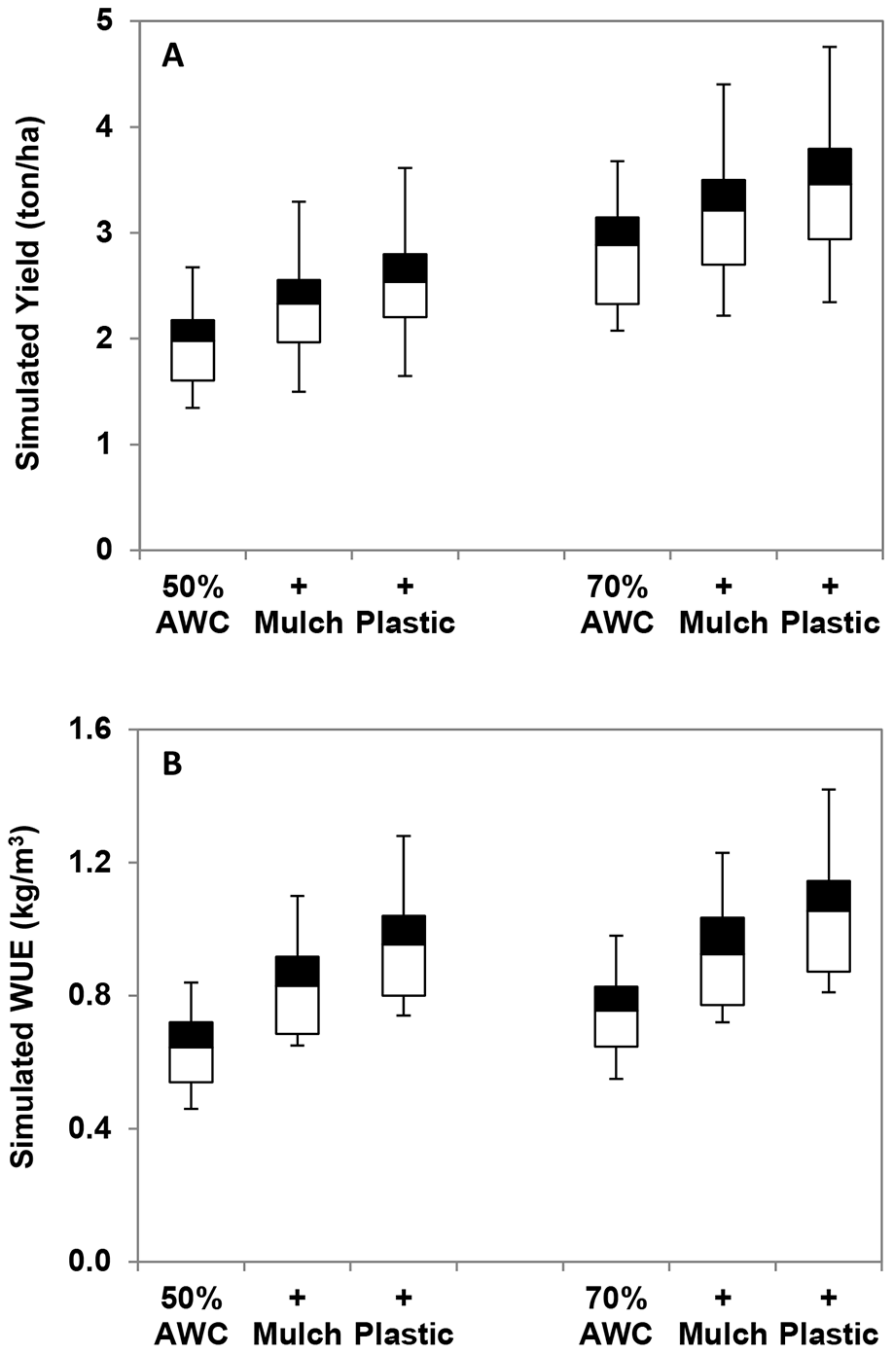
Results of six model experiments; effects of straw mulch and plastic film on wheat yield (A) and water use efficiency (B). Experiment E1: Planting at 50% of FC; Experiment E2: E1+organic mulch; Experiment E3: E1+plastic cover; Experiment E4: Planting at 70% of FC; Experiment E5: E4+organic mulch; Experiment E6: E4+plastic cover.

In model experiment E1 (winter wheat planted at 50% of FC), mean wheat yield and WUE were 2 ton/ha and 0.6 kg/m^3^, respectively (Figure 6). In model experiment E2, with organic mulch during the growing season, mean yield increased to 2.3 ton/ha and WUE increased to 0.8 kg/m^3^. In model experiment E3, with plastic film during the growing season, mean yield increased further to 2.5 ton/ha and WUE to 1.0 kg/m^3^.

Similarly, in model experiment E4 (winter wheat planted at 70% of FC), mean yield and WUE were 2.9 ton/ha and 0.8 kg/m^3^, respectively. With organic mulch (E5), mean yield and WUE increased to 3.2 ton/ha and 0.9 kg/m^3^, and with plastic film cover (E6), mean yield and WUE increased further to 3.5 ton/ha and 1.1 kg/m^3^. Our model experiments show that both organic mulch and plastic film cover could significantly improve yield and WUE, but the impact of plastic cover was bigger than the organic mulch mainly due to high effectiveness in reducing soil evaporation. Moreover, increasing water storage in the soil during the fallow season, so that available soil water content increases from 50 to 70% of FC in autumn at the time of winter wheat seeding, is at least as effective as a plastic cover during the growing season.

## Discussion

We successfully calibrated and validated the AquaCrop model on the basis of the long-term monitoring data (30 years) of rainfed winter wheat on the Loess Plateau of northern China. The full set of crop parameters (Table 3) may provide also guidance to future studies and further model calibrations and validations. We also quantified four key components of the water balance, i.e., rainfall, changes in soil water, soil evaporation and crop transpiration, for both fallow and growing seasons by combining empirical data from a long-term wheat monitoring site with calculated results using the AquaCrop model. In the end, we also explored the potential for increasing wheat yield and WUE through model experiments.

The relationship between observed and modelled wheat yield had a R^2^ of 0.77, slope of 0.9 and an index of agreement (d) of 0.93 (Figure 3). Mkhabela and Bullock [47] reported a R^2^ of 0.66, slope of 0.96, index of agreement (d) of 0.99 between observed and modelled wheat yields. Araya et al. [48] reported a R^2^>0.80 when simulating barley biomass and grain yield. Stricevic et al. [49] reported a R^2^>0.84 when simulating yields of maize, sunflower and sugar beet. Similarly for simulating soil water content, we found a R^2^ of 0.78, slope of 0.9 and an index of agreement (d) of 0.93 (Figure 3). Mkhabela and Bullock [47] reported a R^2^ of 0.9 and a slope of 0.9 for simulating soil water content. Hence, the performance of AquaCrop for our dryland wheat field is largely comparable with that of other modelling studies.

Water stress limited the crop yield at this site. According to our model simulations, water stress has led to suboptimal yields in essentially all years, for both low and relatively high wheat yields. For example, a very low grain yield (0.6 ton/ha) was recorded in the year 2000, when water stress for leaf expansion and stomatal closure started already at the 54^th^ day after planting. In contrast, water stress for leaf expansion and stomatal closure started to occur only from day 156 after planting in 2003, when grain yield was 3.8 ton/ha. In both cases, water stress occurred before flowering stage (∼200 days after planting). Water stress leads to low grain yield and low WUE, but depending on the stage and duration of the water stress [17], [50].

We found significant linear relationships between wheat yield and ET, and between wheat yield and T (Figure 5), in line with some other studies [17], [51], [52]. Mean T/ET ratio in our study was only 58% during the growing season, which was 8–12% lower than that reported by Liu et al. [53] and Kang et al. [54] but highly in line with that reported by Wang et al. [55]. The higher T/ET ratio in the studies of Liu et al. [53] and Kang et al. [54] was probably due to the irrigation treatments where the crop had more water for transpiration. It is well-known that crop yield and WUE are often lower in rainfed agriculture than in irrigated agriculture [17], [20], [50], but depending also on possible nutrient, weed, and disease stresses and irrigation management.

Advanced technologies, such as precision irrigation, are for a long time available but unfortunately not affordable and applicable to the farmers of the Loess Plateau, mainly because of the high cost relative to the low value of cereals [56]. Therefore, we focused on low-cost options, such as straw mulch and plastic film cover because those are the most accessible and low cost materials for farmers to implement in the field. Minimizing soil evaporation could save water for crop transpiration, and thereby increase wheat yield and WUE. Our model experiments suggest that wheat yield can be improved significantly by minimizing soil evaporation via organic mulch and plastic film cover, especially also during the fallow period. Mulching with crop residues can decrease soil evaporation and increase soil water retention. Plastic film cover can significantly increase crop yield and WUE, and promote crop growth during early growth when temperature is low. Our results show that crop yields can be increased by ∼0.9 ton/ha through increasing soil water storage during the fallow period. Crop yields can be increased further by on average ∼0.3 ton/ha through straw mulch and by ∼0.5 ton/ha on through plastic film covers during the growing season. At the same time, WUE increases on average by 0.2 to 0.6 kg/m^3^. These results are in line with results reported by Deng et al. [57] and others [58]–[60].

Evidently, increased rainwater harvesting during the fallow season is an effective option. Straw mulch significantly reduces the evaporative water losses during the fallow season and is conducive to the infiltration of rain water in the soil. Plastic film covers are less applicable during the fallow season, because they may limit the infiltration of rain water and thereby increase runoff. Reduced tillage can also improve soil water storage. According to a recent study of Hou et al. [61], rotational tillage (rotation of no-tillage and subsoiling) could significantly increase soil water storage during the summer fallow and wheat growing season compared with conventional tillage. They found that rotational tillage increased wheat yields by 10%, and WUE by 7.5%, respectively.

## Conclusions

Low wheat yield at the monitoring site was largely due to (i) limited rainfall, (ii) low soil water storage during fallow season because of high water loss via soil evaporation, and (iii) the poor synchronisation of the wheat growing season to the rainfall distribution season. Although water was limited, on average only one-third of the total available water was actually used by the crop for transpiration. The remaining two-thirds was lost by soil evaporation, 60% during the fallow season and 40% during the growing season. Our model experiments suggest that minimizing soil evaporation via organic mulch or plastic film covers can significantly increase wheat yield and WUE. More importantly, these increases can be realized by the application of relatively low cost measures. Further studies are needed to test the effectiveness of these measures in the field.
